# Imipramine impedes glioma progression by inhibiting YAP as a Hippo pathway independent manner and synergizes with temozolomide

**DOI:** 10.1111/jcmm.16874

**Published:** 2021-09-01

**Authors:** Yan Wang, Xiang Wang, Xu Wang, Di Wu, Ji Qi, Yu Zhang, Kai Wang, Ding Zhou, Qing‐Ming Meng, Er Nie, Qiang Wang, Ru‐Tong Yu, Xiu‐Ping Zhou

**Affiliations:** ^1^ Insititute of Nervous System Diseases Xuzhou Medical University Xuzhou China; ^2^ Department of Neurosurgery The Affiliated Hospital of Xuzhou Medical University Xuzhou China; ^3^ Pathological Diagnosis Center Xuzhou Central Hospital Xuzhou China

**Keywords:** glioma, imipramine, proliferation, temozolomide, YAP

## Abstract

Patients with malignant glioma often suffered from depression, which leads to an increased risk of detrimental outcomes. Imipramine, an FDA‐approved tricyclic antidepressant, has been commonly used to relieve depressive symptoms in the clinic. Recently, imipramine has been reported to participate in the suppression of tumour progression in several human cancers, including prostate cancer, colon cancer and lymphomas. However, the effect of imipramine on malignant glioma is largely unclear. Here, we show that imipramine significantly retarded proliferation of immortalized and primary glioma cells. Mechanistically, imipramine suppressed tumour proliferation by inhibiting yes‐associated protein (YAP), a recognized oncogene in glioma, independent of Hippo pathway. In addition to inhibiting YAP transcription, imipramine also promoted the subcellular translocation of YAP from nucleus into cytoplasm. Consistently, imipramine administration significantly reduced orthotopic tumour progression and prolonged survival of tumour‐bearing mice. Moreover, exogenous overexpression of YAP partially restored the inhibitory effect of imipramine on glioma progression. Most importantly, compared with imipramine or temozolomide (TMZ) monotherapy, combination therapy with imipramine and TMZ exhibited enhanced inhibitory effect on glioma growth both *in vitro* and *in vivo*, suggesting the synergism of both agents. In conclusion, we found that tricyclic antidepressant imipramine impedes glioma progression by inhibiting YAP. In addition, combination therapy with imipramine and TMZ may potentially serve as promising anti‐glioma regimens, thus predicting a broad prospect of clinical application.

## 1 INTRODUCTION

Glioblastoma (GBM) is the most common and fatal primary brain tumour in adults, with a median survival of approximately 15 months.^1^, ^2^ The clinical first‐line medication for GBM is temozolomide (TMZ), which have shown limited benefits mainly due to drug insensitivity or acquired resistance.^3^ Therefore, it is urgent to explore new chemotherapeutic agents or repurpose the old clinical used drugs to treat GBM.

Nowadays, psychological depression is usually found to correlate with a poorer clinical outcome in cancer patients. Thus, antidepressants are widely used in tumour patients suffered with moderate to severe depression.^4^, ^5^ Emerging evidence indicates that certain types of antidepressants have anti‐tumour properties in solid tumours apart from their intrinsic antidepressant effects.^6^, ^7^, ^8^ Imipramine, a tricyclic antidepressant (TCA) that functions by inhibiting serotonin and norepinephrine reuptake in the central nervous system (CNS),^9^, ^10^ is currently considered to possess anti‐tumour ability in various non‐CNS tumours, such as small cell lung cancer, prostate cancer, colorectal cancer and lymphomas.^11^, ^12^, ^13^ However, there are few studies to explore the influence of imipramine on glioma and the results provided by previous studies were inconsistent and controversial. For example, Levkovitz et al. reported that imipramine did not induce apoptosis, while Jeon et al. found that imipramine enhanced autophagic and apoptotic activities of glioma cells.^10^, ^14^, ^15^ In addition, the specific role of imipramine, especially the mechanism that affects indefinite proliferation and invasion ability of glioma cells, has not been fully elucidated.

Yes‐associated protein (YAP), the core effector of Hippo kinase cascade, usually plays a significant role in promoting cancer in most human tumours.^16^, ^17^ When Hippo kinase cascade is on, the upstream kinase LATS phosphorylates YAP, making it sequester in the cytoplasm and unable to enter the nucleus to initiate the transcription of downstream target genes. On the contrary, when Hippo kinase cascade is off, YAP enters the nucleus and interacts with the TEA domain (TEAD) family transcription factors, leading to expression of the downstream target genes and tumour growth.^18^, ^19^ Previous investigations performed by others and our group have found that YAP is closely related to the malignant progression of gliomas, and thus, targeting YAP may be helpful for the molecular therapy of glioma.^19^, ^20^, ^21^, ^22^ Notably, recent studies have shown that nortriptyline, another classical TCA, could increase the sphingolipid ceramide through inhibition of acid ceramidase (the enzyme responsible for ceramide metabolism), thus inhibiting YAP signalling in hepatic stellate cells.^23^, ^24^ We therefore wonder whether imipramine will affect the activity of YAP.

In the present study, we investigated the effects of imipramine on glioma cell proliferation and glioma growth by conducting a serial *in vitro* and *in vivo* experiments. In addition, we elucidated the underlying molecular mechanism responsible for proliferation inhibition and evaluated the combinational effect of imipramine and TMZ. Our findings highlighted the importance of YAP as a potential molecular target and uncovered imipramine as a promising drug candidate for glioma therapy. Furthermore, imipramine may be a potential TMZ sensitizer and glioma patients may benefit from imipramine and TMZ combination therapy.

## 2 MATERIALS AND METHODS

### 2.1 Cell lines, antibodies, reagents and plasmids

Glioma cell lines (U87, U251, U373, LN229, GL261) and normal human astrocyte (NHA) were purchased from Shanghai Cell bank, Type Culture Collection Committee, Chinese Academy of Sciences. Primary glioma cell lines (GBM) were established by our laboratory as previously described.^25^ All glioma cells were cultured in DMEM supplemented with 10% foetal bovine serum (FBS). Normal human astrocyte was cultured in astrocyte medium (ScienCell; Cat No.1801) supplemented with penicillin/streptomycin, 2% FBS and astrocyte growth supplement. All cells were maintained in humidified incubator with 5% CO2 at 37°C.

The anti‐YAP, anti‐p‐YAP (Ser127), anti‐MST, anti‐p‐MST (Thr183), anti‐LATS, anti‐p‐LATS (Ser909) and anti‐GAPDH primary antibodies were obtained from Cell Signaling Technology. Anti‐CYR61 and anti‐CTGF antibodies were purchased from Santa Cruz Biotechnology. Imipramine (IMIP) and temozolomide (TMZ) were purchased from TargetMol. Control and YAP wild‐type (WT) plasmids were kindly gifted by Prof. Hongbin Ji at the Institute of Biochemistry and Cell Biology, Shanghai Institutes for Biological Sciences, Chinese Academy of Sciences.^26^

### 2.2 Establishment of YAP‐overexpression stable cells

Stable YAP‐overexpressing U251 and GBM cells were generated by a lentiviral‐based approach, which have been described previously.^20^

### 2.3 Cell counting kit‐8 assay

Cell viability and half‐maximal inhibitory concentration (IC50) values were determined using cell counting kit‐8 (CCK8) assay according to the manufacturer’s protocol as previously described.^20^

### 2.4 Colony formation assay

The proliferation ability of cells was detected using six‐well plates described previously.^20^ Briefly, a total of 1000 cells were seeded into each well followed by imipramine treatment for 48 h and cultured at 37℃ for 2 weeks to form colonies. Cells were then gently washed with PBS, fixed with 4% paraformaldehyde and stained with 0.03% crystal violet. The plates were dried at room temperature, and the number of colonies was counted.

### 2.5 EdU incorporation assay

Following the manufacturer’s instructions, we used the Cell‐Light EdU Cell Proliferation Detection Kit (Ruibo Biotech) for EdU incorporation assay as previously reported.^20^

### 2.6 Transwell invasion assay

Cell invasion assay was conducted using a transwell system that incorporated a polycarbonate filter membrane with a diameter of 6.5 mm and pore size of 8 μm (Corning, NY), according to the manufacturer’s instruction and previous study.^27^ Briefly, transwell membranes were precoated with DMEM‑diluted Matrigel® (BD Biosciences) for 3 h at 37°C. Cells (8 × 10^3^) were plated in the upper chambers of transwell plates in 200 µl serum‑free culture DMEM medium. A total of 500 µl DMEM medium supplemented with 10% FBS was plated in the lower chambers. Following incubation for 48 h after imipramine treatment, the invasive cells were fixed in methanol for 15 min and subsequently stained for 15 min at room temperature with 0.1% crystal violet. The invasive cells were counted in five random microscopic fields each chamber.

### 2.7 Subcellular fractionation and Western blotting

Cellular fractionation was performed using a Membrane and Cytosol Protein Extraction Kit (Beyotime) following manufacturer’s instructions. Protein concentration was detected using a BCA protein assay kit (Beyotime). Thereafter, equal amounts of protein were separated by 10% sodium dodecyl sulphate polyacrylamide gel electrophoresis (SDS‐PAGE) and transferred to 0.45 μm pore size PVDF membrane (Roche). After blocking for 2 h with 3% BSA, membranes were incubated with primary antibodies at 4°C overnight, then with secondary antibodies at room temperature for 2 h. Finally, the immunoreactive proteins were visualized using enhanced chemiluminescence and protein bands were measured with ImageJ software.

### 2.8 RNA extraction and qRT‐PCR

Total RNA was extracted from cultured cell lines with Trizol (Invitrogen), followed by synthesis of first‐strand cDNA using a reverse transcription kit (Tiangen). Quantitative RT‐PCR was performed with SuperReal PreMix Plus (Tiangen) according to the manufacturer’s instructions. The relative mRNA expression levels of target genes were normalized to the GAPDH internal control and calculated with the $2^{‐\Delta \Delta C_{t}}$ method. All primer sequences were synthesized by Sangon Biotech Co. and listed in Table 1.

**TABLE 1:** Primers used for the real‐time PCR analysis

Gene name	Primer sequence (Forward 5′ to 3′)	Primer sequence (Reverse 5′ to 3′)
YAP	CACAGCTCAGCATCTTCGAC	TATTCTGCTGCACTGGTGGA
LATS	ACTCACAGACAGATGTAGGA	GAGAGGTGGTGGAGGATAGC
MST	ACAAATCCTCCTCCCACATTCCG	CACTCCTGACAAATGGGTGCTG
CTGF	AGGAGTGGGTGTGTGACGA	CCAGGCAGTTGGCTCTAATC
CYR61	CCTTGTGGACAGCCAGTGTA	ACTTGGGCCGGTATTTCTTC
GAPDH	GCACCGTCAAGGCTGAGAAC	TGGTGAAGACGCCAGTGGA

### 2.9 Immunofluorescence

The subcellular localization of YAP in tumour cells was detected using immunofluorescence assay. All steps were performed as described previously.^20^

### 2.10 Patient‐derived xenograft and intracranial tumour mouse models

All the *in vivo* experiments performed in this study were approved by the Institutional Ethics Committee of Xuzhou Medical University. For the YAP restoring experiment, luciferase‐GFP‐YAP or luciferase‐GFP‐vector GBM cells (5 × 10^5^) were injected into BALB/c male nude mice (4 weeks, 20 g) intracranially following with imipramine (20 mg/kg) or vehicle 5 days on, 2 days off for 3 weeks. For the synergism experiment, BALB/c nude mice or C57BL/6 mice were intracranially injected with luciferase‐GFP‐GBM cells (5 × 10^5^) or luciferase‐mCherry‐GL261 cells (2 × 10^5^), respectively. After transplantation, the mice were administered with imipramine (20 mg/kg) or TMZ (7.5 mg/kg) intraperitoneally alone or together with imipramine (20 mg/kg) 5 days on, 2 days off for 3 weeks. Bioluminescence imaging was used to detect intracranial tumour growth on day 7, day 14 and day 21. After the tumour‐bearing mice exhibited hemiplegia, listlessness, cachexia and other neurological symptoms, they were anesthetized with 5% isoflurane for 90 s and euthanized by cervical dislocation. Then, the main organs (including heart, liver, spleen, lung, kidney and glioma‐bearing brain) were removed for subsequent experiments. Kaplan‐Meier curves were calculated to estimate the overall survival.

### 2.11 RNA sequencing and screening of differentially expressed genes

RNA library preparation and sequencing analysis of DMSO and imipramine (20 μM)‐treated U251 cells were conducted using BGISEQ‐500 platform (Wuhan, China). Statistical analysis was performed, and differentially expressed genes (DEGs) were screened based on *Q*‐value and fold change using NOISeq method.^28^, ^29^ Kyoto Encyclopedia of Genes and Genomes (KEGG) pathway were applied for the analysis of DEGs using the Dr. Tom online software (BGI). *Q*‐value of the pathway shown in the figure was <0.05.

### 2.12 Statistical analysis

All experiments were repeated at least three times and presented as the mean ± SEM. Significant differences within groups were analysed using Student’s *t* test for single comparisons and one‐way ANOVA followed by Tukey’s test for multiple comparisons using GraphPad Prism 6 software. Overall survival curves were calculated using the Kaplan‐Meier method and compared using the log‐rank test. *p* Values <.05 were considered be statistically significant (*p < .05, **p < .01, ***p < .001).

## 3 RESULTS

### 3.1 Imipramine inhibits the proliferation and invasion of glioma cells

**FIGURE 1:**
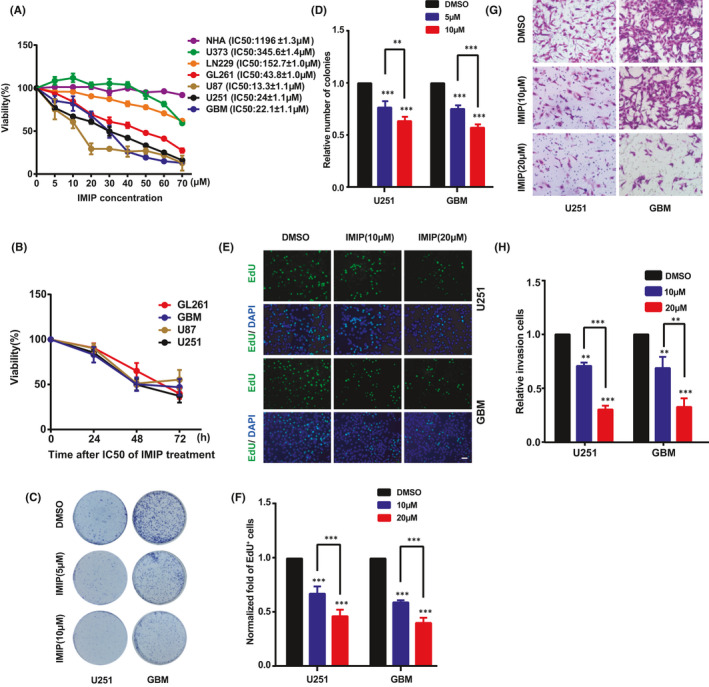
Imipramine suppresses glioma cell proliferation and invasion. A, CCK‐8 cell viability assay of six glioma cell lines and one normal human astrocyte cell line treated with different concentrations of imipramine, including 0, 10, 20, 30, 40, 50, 60 and 70 μM for 48 h. B, CCK‐8 assay of four sensitive glioma cell lines treated with respective IC50 of imipramine for 72 h. C&D. Representative images (C) and quantitative results (D) of colony formation assay after U251 and GBM cells having been treated with indicated concentration of imipramine. E,F, Representative images (E) and quantitative results (F) of EdU assay in U251 and GBM cells treated with indicated concentration of imipramine, scale bar: 50 μm. G,H, Representative images (G) and quantitative results (H) of invasive cells after indicated concentration of imipramine treatment, scale bar: 50 μm. Data were mean ± SEM for the three replicates. *p < .05, **p < .01, ***p < .001

To evaluate the effect of imipramine on glioma cell viability, we adopted CCK‐8 assay in six glioma cell lines. The results indicated that imipramine inhibited the viability of glioma cells in a dose‐dependent manner in most tested cell lines. The respective half‐maximal inhibitory concentration (IC50) values differed as the sensitivity of the cells to imipramine varied (Figure 1A). Intriguingly, there was almost no effect on NHAs at the concentrations tested, suggesting high specificity of imipramine to glioma cells (Figure 1A). Moreover, four imipramine‐sensitive cell lines (with low IC50) exhibited cell proliferation inhibition in a time‐dependent manner, especially in U251 and GBM primary cells (Figure 1B). We thus chose U251 and GBM cells as representative cell lines for all following experiments. As shown in Figure 1C,D, compared to the vehicle (DMSO)‐treated cells, imipramine (10 μM) treatment markedly inhibited the colony formation ability in both U251 and GBM cells. The number of colonies formed by U251 cells treated with 10 μΜ imipramine decreased to 63.51%, and similar results were observed in GBM cells. EdU incorporation assay also showed that a significant decrease in EdU‐positive cells was detected in imipramine‐treated cells (Figure 1E,F). The U251 and GBM cells treated with 20 μM imipramine showed a reduction in the EdU‐positive rate to 46.25% and 40.05%, respectively. Since invasiveness has always been considered as a vital biological characteristic of malignant glioma cells, we also investigated the effect of imipramine on glioma cells invasion. The results indicated that the invasive ability of U251 and GBM cells was significantly inhibited by imipramine (Figure 1G). As shown in Figure 1H, cell invasion was, respectively, inhibited to 30.32% and 32.67% after 20 μM imipramine treatment in both cells. Taken together, these results indicate that imipramine significantly suppresses proliferation and invasion ability of glioma cells.

### 3.2 Imipramine inhibits YAP activity as a Hippo pathway independent manner

**FIGURE 2:**
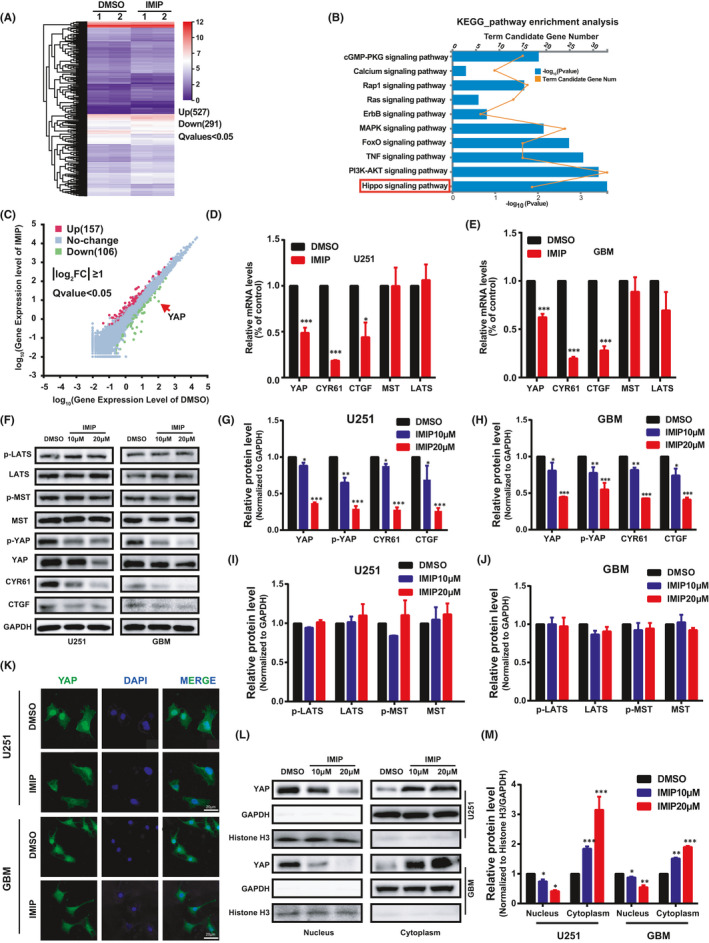
Imipramine inhibits YAP level and nuclear localization independent of activating Hippo pathway. A, Heat map showing all 818 differentially expressed genes (DEG) between two imipramine‐treated cells (IMIP‐1, 2) and two DMSO‐treated cells (DMSO‐1, 2). Gradient colour barcode indicated fold change of expression (log_2_). B, KEGG pathway enrichment analysis of the DEGs in imipramine‐treated cells vs. DMSO‐treated cells. The top 10 enriched pathways were shown. C, Scatter plots of all expressed genes. Red and green indicated upregulated or downregulated genes after imipramine treatment. The DEG is defined as that with FDR ≤0.001 and abs(log2(FC)) ≥1. D,E. Relative mRNA expression levels of Hippo pathway related genes as determined, respectively, by real‐time PCR assay after imipramine treatment in U251 and GBM cells. F, The levels of Hippo pathway‐related proteins in U251 and GBM cells with different doses of imipramine treatment. G–J, Quantitative analysis of the results in (F). K, Representative confocal images of YAP subcellular localization immunofluorescence (green) after imipramine treatment. Scale bar: 20 μm. L, Subcellular localization of YAP was examined by using cellular fractionation after imipramine treatment. Histone H3 and GAPDH were used as nucleus and cytoplasm loading control, respectively. M, Quantitative analysis of the results in (L). Data were mean ± SEM for the three replicates. *p < .05, **p < .01, ***p < .001

To investigate the molecular mechanism by which imipramine inhibits the proliferation of glioma cells, we performed high‐throughput RNA‐sequencing (RNA‐seq) analysis to screen differentially expressed genes (DEGs) between DMSO and imipramine‐treated cells. As a result, the DEGs analysis showed that 527 genes were up‐regulated and 291 genes were down‐regulated in imipramine‐treated group (Figure 2A). The KEGG annotation and pathway enrichment analysis illustrated that, of these DEGs, 192 genes were annotated and enriched in ‘Environmental Information Processing’, including signal transduction and signalling molecules and interaction (Figure S1). Next, we analysed the top ranked 10 signal transduction pathways among the aforementioned KEGG pathway terms and found that these DEGs were ordinally and significantly associated with Hippo signalling pathway, PI3K‐Akt signalling pathway, TNF signalling pathway, FoxO signalling pathway and so on (Figure 2B). DEGs were further illustrated in the scatter‐plot (|log_2_fold change|≥1.0 and FDR ≤ 0.001). To our surprise, YAP, the pivotal transcriptional co‐activator of the Hippo pathway, was significantly down regulated after imipramine treatment (Figure 2C). YAP has been reported to play vital functions in controlling organ size by regulating cell proliferation and survival, especially in tumour cells.^30^, ^31^ We therefore chose YAP as the most likely candidate gene mediating the inhibitory effect of imipramine on glioma progression.

To validate the results of RNA‐Seq, we performed qRT‐PCR to determine the level of YAP after imipramine treatment. As shown in Figure 2D,E, the mRNA level of YAP and its target genes CYR61 and CTGF decreased significantly in imipramine treated group in both U251 and GBM cells. Consistently, Western blot analysis also showed a substantial reduction in YAP total and p‐YAP level, as well as those of CYR61 and CTGF, after 20 μM imipramine treatment in both cells (Figure 2F–H). Since the sensitivity to imipramine treatment of different cell lines clearly differed (Figure 1A), we sought to revisit this result and found that endogenous YAP level in these cells were strongly correlated with the sensitivity to imipramine (Figure S2). As a transcriptional co‐activator, the function of YAP is strictly constrained by its subcellular localization, which prompted us to further examine the effect of imipramine on YAP distribution using immunofluorescence assay. As shown in Figure 2K, compared with the DMSO treated group, YAP nuclear level decreased, while increased in cytoplasm after imipramine treatment. Furthermore, by using cellular fractionation and immunoblotting, we got similar results in both cells (Figure 2L,M).

Since YAP is a key effector molecule downstream of Hippo signalling pathway,^32^, ^33^ we wondered whether the regulation of YAP by imipramine is Hippo‐dependent. Therefore, both the mRNA and protein levels of YAP direct upstream kinases, such as MST and LATS, were determined by qRT‐PCR and Western blot analysis after imipramine treatment. As presented in Figure 2F,I,J, no significant changes in MST or LATS total and phosphorylation levels were observed. These findings demonstrate that imipramine decreases YAP protein level and nucleus translocation independent of Hippo pathway activation, thereby blocking the downstream effectors and ultimately weakening glioma cell proliferation.

### 3.3 The effect of imipramine on glioma progression was partially mediated by YAP

**FIGURE 3:**
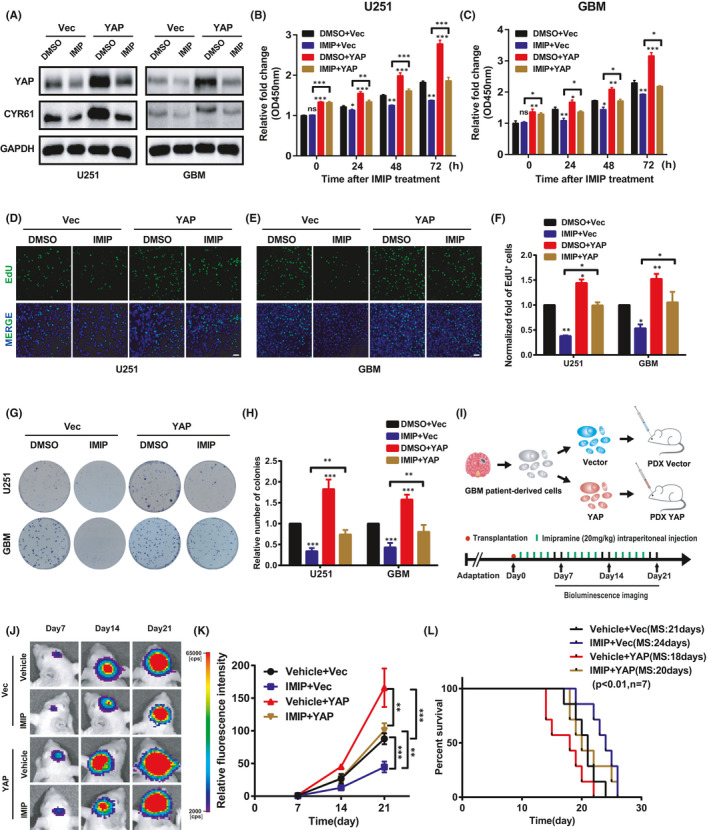
YAP partially mediated proliferation and invasion inhibition of imipramine on glioma cells. A, YAP and CYR61 protein levels were restored by YAP overexpression after imipramine treatment using Western blotting. B,C, CCK‐8 assay shows that the inhibition effects of imipramine on U251 and GBM cells were restored by YAP‐overexpression. D,E, EdU assay was performed to assess cell proliferation in YAP‐overexpression cells after imipramine treatment in U251 and GBM cells. Scale bar: 50 μm. F, Statistical results for the EdU assay in (D,E). G, Representative images of colony formation assay after treatment with imipramine in YAP‐overexpression U251 and GBM cells. H, Quantitative analysis of the results in (G). I, Schematic representation of the PDX xenograft experimental workflow. J, Representative bioluminescence images of intracranial xenografts bearing YAP‐overexpression or control cells followed by imipramine administration on the indicated days. K, Quantitative analysis of the fluorescence index. L, Kaplan‐Meier analysis of the median survival time of mice. Data were mean ± SEM for the three replicates. *p < .05, **p < .01, ***p < .001

Based on the above data, we hypothesized that YAP may participate in the inhibition effect of imipramine on glioma cell proliferation and invasion. To address this question, we constructed U251 and GBM cell lines stably expressing wide type YAP and followed by imipramine treatment with indicated concentrations. As shown in Figure 3A, the decreased level of YAP and CYR61 induced by imipramine treatment were partially restored by exogenous YAP. Thereafter, CCK‐8 assay revealed that overexpression of YAP significantly promoted the proliferation of U251 and GBM cells and partially abolished the inhibitory effect of imipramine on glioma cell growth at different time points (Figure 3B,C). Consistently, the results of EdU incorporation (Figure 3D–F), colony formation (Figure 3G,H) and transwell invasion assays (Figure S3) showed that YAP overexpression partially cancelled the suppression of imipramine on glioma cell proliferation and invasion. In addition, we established patient‐derived xenograft (PDX) model in nude mice to further investigate the effects of imipramine on glioma growth *in vivo* (Figure 3I). As the bioluminescence results presented in Figure 3J,K, tumour growth was significantly slowed down over time in the imipramine treated group, while increased in YAP overexpression group. Furthermore, YAP overexpression abolished the inhibition effect of imipramine on tumour growth. In agreement with this, Kaplan‐Meier analysis showed that, even after imipramine treatment, the survival time of mice bearing tumours from YAP overexpression cells was significantly shortened (Figure 3L). Overall, these data indicate that up‐regulation of YAP blocks the inhibitory effect of imipramine on glioma growth both *in vitro* and *in vivo*.

### 3.4 Combining imipramine with TMZ attenuated glioma cell proliferation *in vitro* and the growth of glioma *in vivo*

**FIGURE 4:**
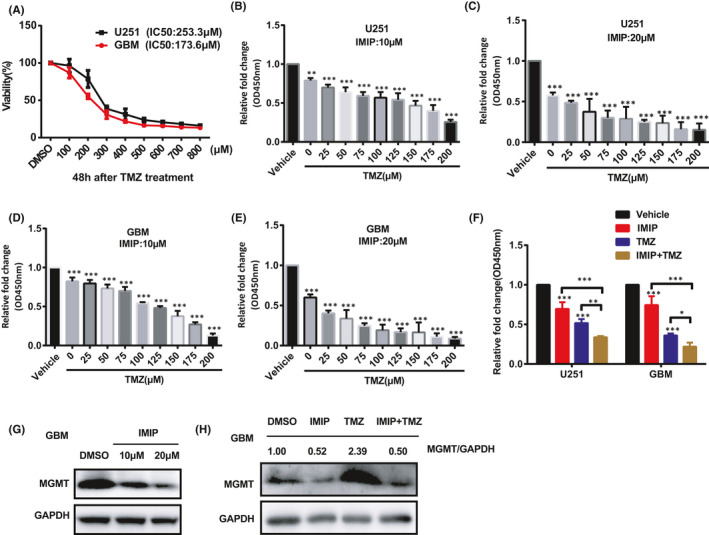
Imipramine enhanced the proliferation inhibition effect of TMZ on glioma cells *in vitro*. A, CCK‐8 assay showing the viability of U251 and GBM cells treated with different concentrations of temozolomide, including 0, 100, 200, 300, 400, 500, 600, 700 and 800 μM for 48 h. B–E, Cells were treated with DMSO only (Vehicle) or increasing doses of TMZ with 10 μM (B,D) or 20 μM (C,E) imipramine for 48 h. Relative proliferation of U251 and GBM cells treated with imipramine and indicated doses of TMZ were determined by CCK‐8 assay. F, Cells were treated with vehicle, imipramine (10 μM), TMZ (150 μM) or imipramine plus TMZ, respectively, for 48 h. Cell viability was detected using the CCK‐8 assay. G, The MGMT protein levels in the GBM cell after different doses of imipramine treatment. H, Increased MGMT protein levels by TMZ treatment were suppressed after the combination treatment. Data were mean ± SEM for the three replicates. *p < .05, **p < .01, ***p < .001

It is well documented that chemotherapy is a critical process in the postsurgical treatment of glioma.^34^ Since imipramine exhibits the significant inhibitory effect of glioma cell proliferation in this study and processes the ability to penetrate the blood–brain barrier,^35^ we wonder whether it could synergize with TMZ, the first‐line drug in treating malignant glioma. Firstly, U251 and GBM cells were treated respectively with TMZ for 48 h and IC50 was determined by CCK8 assay. As shown in Figure 4A, the IC50 values of U251 and GBM cells were 253.3 and 173.6 μM, respectively. To evaluate the effect of imipramine combined with TMZ on glioma cell proliferation, U251 and GBM cells were treated with fixed doses of 10 or 20 μM imipramine, both of which were below their respective IC50, following by different concentrations of TMZ ranging from 0 to 200 μM. As presented in Figure 4B–E, in both cell lines, the cell proliferation rate was reduced with imipramine treatment and it was further inhibited when combined treatment with different concentrations of TMZ. According to the results of CCK8, the appropriate concentration of TMZ was selected to determine the potential synergistic effect with 10 μM imipramine. To our delight, compared with imipramine or TMZ monotherapy, the combination therapy with TMZ and imipramine apparently induced a decline of cell viability of glioma cells. The inhibition of cell proliferation was increased by 17.92% and 14.14% in the combined group, compared to the TMZ alone group in U251 and GBM cells, respectively. Similarly, the inhibition rate was increased by 35.72% and 52.47% when comparing with the imipramine alone group in both cells (Figure 4F). The concentration of synergetic killing effect of imipramine and TMZ was lower than their respective IC50 in TMZ or imipramine treatment alone group. In addition, the combination of both agents exhibited a synergistic effect due to the combination index (CI) calculated by Compusyn.^36^ As illustrated in Tables 2 and 3, the CI values for both cell lines were considerably <0.4 when treated with 20 μM imipramine, indicating a strong synergism of the inhibition effect. Thus, these results indicated that utilizing imipramine may sensitize the inhibition effect of TMZ in glioma cells *in vitro*. Extensive evidence reported that the DNA lesions caused by TMZ could be repaired by O6‐alklguanine DNA alkyltransferase encoded by O‐6‐methylguanine‐DNA methyltransferase (MGMT) gene.^37^, ^38^ Therefore, we wondered whether imipramine affected MGMT level and further examined it in GBM cells. To our surprising, the MGMT protein levels was markedly repressed by imipramine treatment (Figure 4G). Not only that, the elevated MGMT levels after TMZ treatment were significantly inhibited by combined treatment (Figure 4H), implying the possible underlying molecular mechanisms for the synergism.

**TABLE 2:** Combination index (CI) of IMIP and TMZ in U251 cells

IMIP concentration (μM)	TMZ concentration (μM)	Effect of combination	CI value of combination^a^
10	100	0.432	0.65561
10	150	0.535	0.53085
10	200	0.745	0.39201
20	100	0.712	0.23324
20	150	0.764	0.28852
20	200	0.848	0.29546

^a^ CI value <1 indicated that there is a synergistic effect of IMIP combined with TMZ.

**TABLE 3:** Combination index (CI) of IMIP and TMZ in GBM cells

IMIP concentration (μM)	TMZ concentration (μM)	Effect of combination	CI value of combination^a^
10	100	0.457	0.82528
10	150	0.624	0.62570
10	200	0.872	0.31967
20	100	0.808	0.27275
20	150	0.836	0.32204
20	200	0.913	0.25868

^a^ CI value <1 indicated that there is a synergistic effect of IMIP combined with TMZ.

**FIGURE 5:**
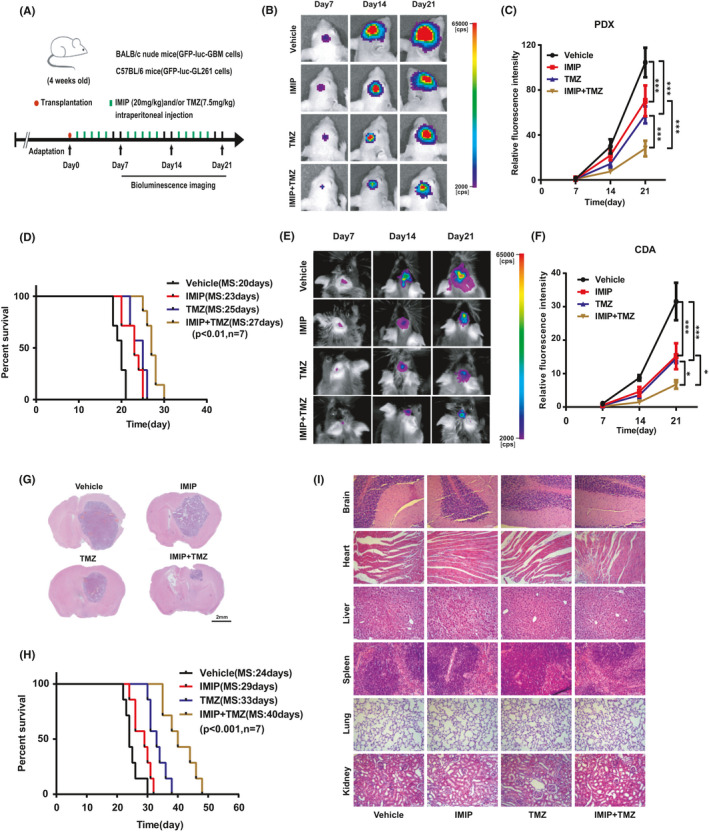
Combining imipramine with TMZ attenuated the growth of glioma *in vivo*. A, Schematic representation of the patient‐derived xenograft (PDX) or cell‐derived allograft (CDA) experimental workflow. B, Representative bioluminescence images of intracranial xenografts bearing luc‐GFP‐GBM cells with different drug administration on the indicated days in nude mice. C, Quantitative analysis of the fluorescence index. D, Kaplan‐Meier analysis of the median survival time of nude mice bearing tumour. E, Representative bioluminescence images of intracranial allografts bearing luc‐mCherry‐GL261 cells with different drugs administration on the indicated days in C57BL/6 mice. F, Quantitative analysis of the fluorescence index. G, Representative images of H&E staining of whole‐brain sections from groups with different drugs administration. H, Kaplan‐Meier analysis of the median survival time of mice. I, H&E staining of brain, heart, liver, spleen, lung and kidney of the tumour‐bearing mice treated with different drugs administration. Scale bar: 100 µm. *p < .05, **p < .01, ***p < .001

To further evaluate the therapeutic efficacy of combining imipramine with TMZ *in vivo*, we performed experiments in PDX models. After the tumour cells transplantation, mice were randomized into four groups to receive vehicle, imipramine, TMZ, or imipramine plus TMZ. The mode of administration and dosing schedule is presented in Figure 5A. We found that either TMZ or imipramine alone significantly delayed tumour growth, while the combined effect of TMZ and imipramine exhibited the highest of tumour growth inhibition (Figure 5B,C). Importantly, imipramine in combination with TMZ significantly extended the median overall survival of tumour‐bearing nude mice compared to either agent alone, consistent with our *in vitro* findings that imipramine enhances the sensitivity of glioma cells to TMZ (Figure 5D).

To rule out the possibility that these observations were due to a specific intracranial tumour mouse model, we conducted this *in vivo* experiment of combination therapy in a murine‐derived tumour model. The results analysis of both bioluminescence imaging (Figure 5E,F) and haematoxylin and eosin (H&E) staining (Figure 5G) showed that the inhibition in glioma growth was considerably more pronounced in imipramine and TMZ combination treatment. Similarly, the combination therapy greatly extended the median survival time of the tumour‐bearing C57BL/6 mice (Figure 5H). These results indicate that imipramine may act as a potent sensitizer for TMZ chemotherapy *in vivo*.

Additionally, we evaluated the histology of major organs including brain, heart, liver, spleen, lung and kidney excised from mice to investigate systemic toxicity (Figure 5I). The results of HE staining indicated that no histopathological changes were observed compared among the groups, suggesting that imipramine and TMZ effectively treats intracranial gliomas without conferring any apparent toxicity to normal tissues.

## 4 DISCUSSION

**FIGURE 6:**
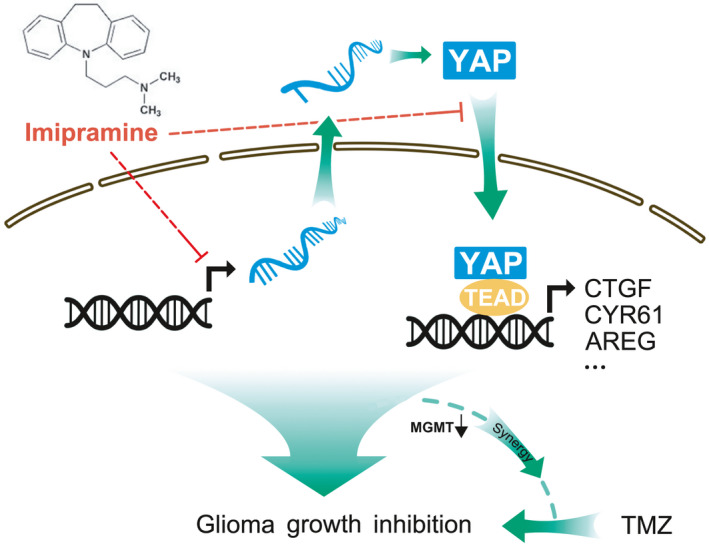
Schematic model of how imipramine inhibits glioma cell proliferation. The results showed that imipramine impedes tumour progression through diminishing YAP activity and sensitives glioma cells to temozolomide

Glioblastoma, the most malignant primary brain tumours, is characterized by the extensive proliferative ability and insensitive to chemotherapeutics, which results in poor clinical outcomes and short survival time.^39^, ^40^ Hence, searching novel and effective drugs for the treatment of GBM is extremely urgent. In this study, we found that imipramine significantly inhibited the proliferation and invasion of glioma cells, as well as the intracranial PDX. Mechanistically, imipramine impedes glioma growth by inhibiting YAP expression and translocation from cytoplasm into nucleus, without affecting Hippo signalling pathway as a prerequisite. More importantly, imipramine is found to be a potent sensitizer for TMZ chemotherapy as combination therapy with TMZ, which exhibits strongly tumour suppressing ability both *in vitro* and *in vivo* (Figure 6).

More than 90% of GBM patients suffer from depressive disorders^41^ and increasing epidemiological studies suggest that antidepressants reduce the cancer risk in glioma patients.^42^ Imipramine, the first identified TCAs, is involved in reuptake of serotonin and norepinephrine, importance of which is increasingly recognized.^42^ Studies have shown that imipramine exhibits inhibition role in several types of tumours although the specific mechanism remained unclear.^11^, ^12^, ^43^ A previous study by Shchors et al. demonstrated that imipramine reduces cell viability and increased survival in glioma‐bearing mice by elevating cAMP levels, a modulator of autophagy.^44^ Furthermore, Hsu et al. reported that imipramine induces apoptosis through inhibiting NF‐κB signalling pathway.^15^ To the best of our knowledge, our present study is the first to explore molecular mechanisms underlying proliferation regulation of imipramine on glioma cells using systematic screening methods. Based on RNA‐seq technology, we identified YAP, a classical oncogene closed to development and progression of tumours, as the direct effector involved in regulation of imipramine on glioma progression.

As the key downstream transcription co‐activator of Hippo signalling pathway, YAP usually plays a role on promoting tumour progression. In the present study, we found that imipramine inhibits YAP expression and translocation from cytoplasm into nucleus and thus retards glioma progression. Interestingly, we discovered that, as shown in Figure 2F,I,J, neither mRNA nor protein levels of MST/LATS were altered, indicating that regulation of YAP levels by imipramine was largely independent of MST/LATS kinase activity. Given that imipramine functions by inhibiting serotonin and norepinephrine reuptake, we hypothesized that the mechanism of imipramine on YAP inhibition may possibly be associated with regulation of serotonin and norepinephrine. Dethlefsen and colleagues have comprehensively shown that norepinephrine leads sequestration of YAP to the cytosol and suppression of downstream genes in breast cancer cells.^45^ In addition, Fang et al. found that serotonin‐pERK‐YAP axis mediates liver regeneration and speculated serotonin as an up‐regulator of YAP.^46^ A recent study has demonstrated that TCAs induce hepatic stellate cell inactivation through increasing the sphingolipid ceramide, which was regard as a potent inhibitor of YAP signaling.^23^ We proposed that important neurotransmitters and enzymes may be involved in the biological modulation process of imipramine on YAP in glioma directly or indirectly. However, the accurate regulation mechanism, especially the regulation of YAP mRNA level by imipramine, remains largely unknown and needs to be further studied.

Being a key therapeutic agent widely utilized to treat GBM patients after surgery, TMZ results in a modest increase in overall survival of patients. It has been confirmed that drugs which prompts increased sensitivity to temozolomide could further improve the prognosis of glioma patients.^47^, ^48^ Based on a series of experiments and the calculated CI values in this study, combination of TMZ with imipramine exhibited significant synergism on the suppression of glioma progression. Growing evidence associates chemotherapy effect of TMZ with regulation of MGMT levels.^49^ Although we failed to detect MGMT protein level in U251 cells, we found it significantly decreased by imipramine in the more representative primary GBM cell, suggesting the possible molecular mechanism for this synergistic effect. In order to ensure the stability and reliability of our results, we performed the combination treatment experiments in BALB/c mice and C57BL/6 mice, simultaneously. The results from both models were consistent. Notably, according to the dose equivalence between human and rodents,^50^ the dose of imipramine (20 mg/kg) administered to treat tumour‐bearing mice in our experiments is lower than the clinical dose (average of 150 mg/day) used for treating patients with depression,^51^ indicating the efficacy and safety of low‐dose imipramine treatment. These compelling preclinical evidences suggested that imipramine may potentially be a monotherapy agent and as combination therapy with TMZ for glioma treatment.

In summary, our results demonstrate that imipramine dampened glioma progression by inhibiting YAP activity independent of activating Hippo pathway. Furthermore, imipramine may serve as a potential TMZ sensitizer and glioma patients with high expression of YAP and obvious depression may benefit from combination therapy.

## CONFLICT OF INTEREST

The authors declare that they have no competing interests.

## AUTHOR CONTRIBUTIONS

**Yan Wang:** Conceptualization (lead); Data curation (lead); Formal analysis (lead); Writing‐original draft (lead). **Xiang Wang:** Data curation (equal); Formal analysis (equal); Investigation (equal); Writing‐original draft (supporting). **Xu Wang:** Data curation (supporting); Formal analysis (supporting); Investigation (equal). **Di Wu:** Resources (lead); Validation (lead); Visualization (supporting). **Ji Qi:** Data curation (supporting); Investigation (equal); Visualization (equal). **Yu Zhang:** Conceptualization (supporting); Investigation (supporting); Validation (supporting). **Kai Wang:** Data curation (supporting); Investigation (supporting); Validation (supporting). **Ding Zhou:** Conceptualization (supporting); Resources (supporting); Visualization (supporting). **Qing‐Ming Meng:** Investigation (supporting); Validation (supporting). **Er Nie:** Conceptualization (supporting); Methodology (lead); Validation (supporting). **Qiang Wang:** Methodology (supporting); Software (supporting). **Ru‐Tong Yu:** Supervision (equal); Writing‐review & editing (equal). **Xiu‐Ping Zhou:** Conceptualization (lead); Supervision (lead); Writing‐review & editing (lead).

## ETHICS APPROVAL AND CONSENT TO PARTICIPATE

This project was examined and verified by Laboratory Animal Ethics Committee of Xuzhou Medical University in accordance with Guide to Laboratory Animal Ethics Examination of Xuzhou Medical University. Relative animal experiments are permitted.

## Supporting information

Fig S1

Fig S2

Fig S3

Supplementary Material
