# Supplementary figures and images for: Imipramine impedes glioma progression by inhibiting YAP as a Hippo pathway independent manner and synergizes with temozolomide
Source: J Cell Mol Med. 2021 Sep 1;25(19):9350–63. doi: 10.1111/jcmm.16874 (PMC8500960; doi:10.1111/jcmm.16874)

Figure S1

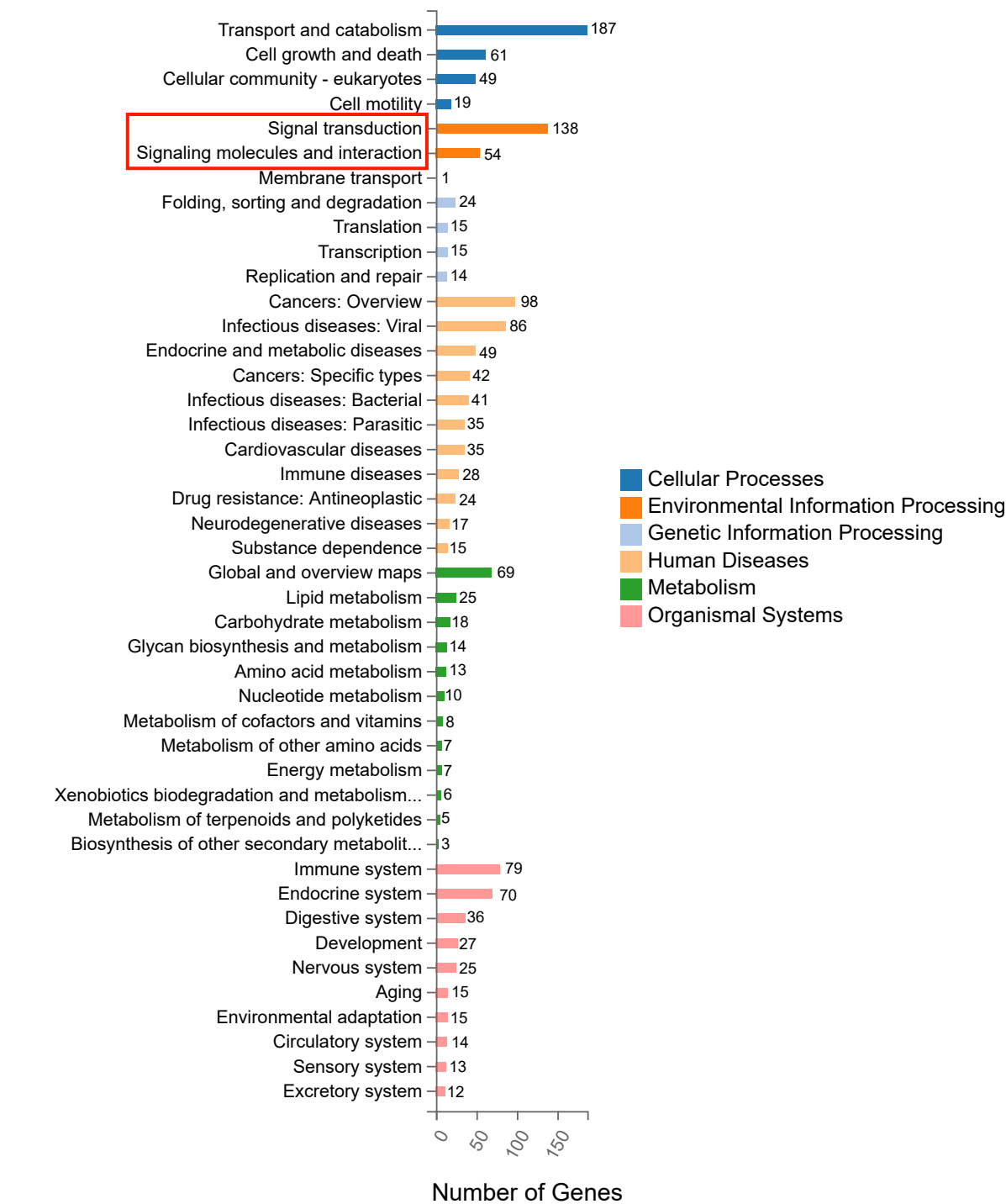

Supplement: Supplementary file 1 — Fig S1 [file JCMM-25-9350-s002.pdf]

Figure S2

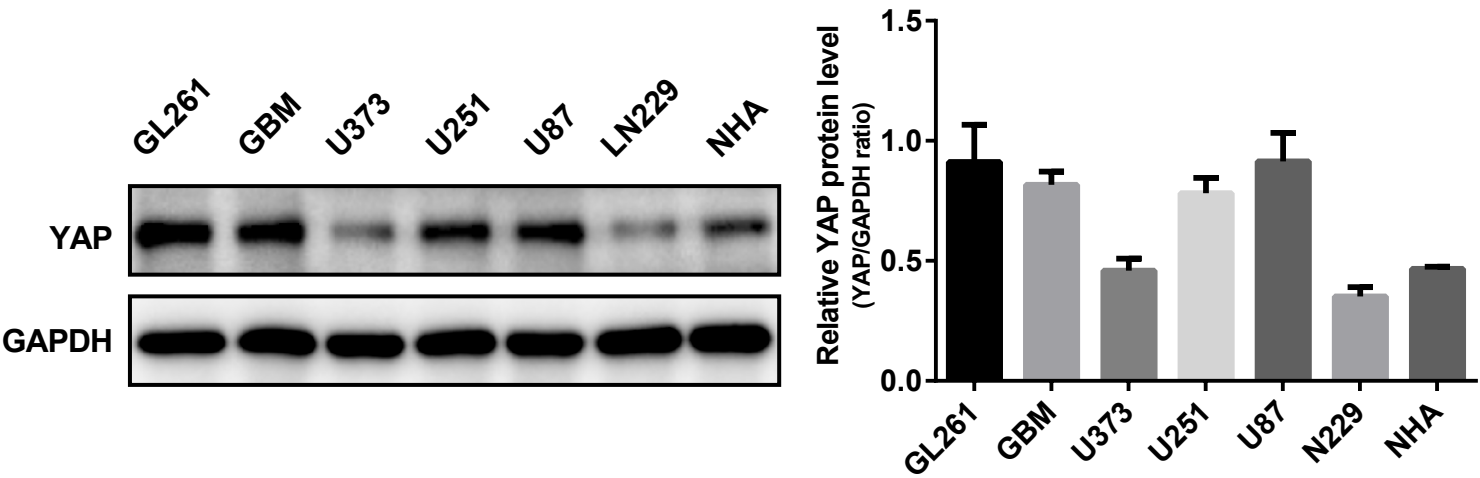

Supplement: Supplementary file 2 — Fig S2 [file JCMM-25-9350-s003.pdf]

Figure S3

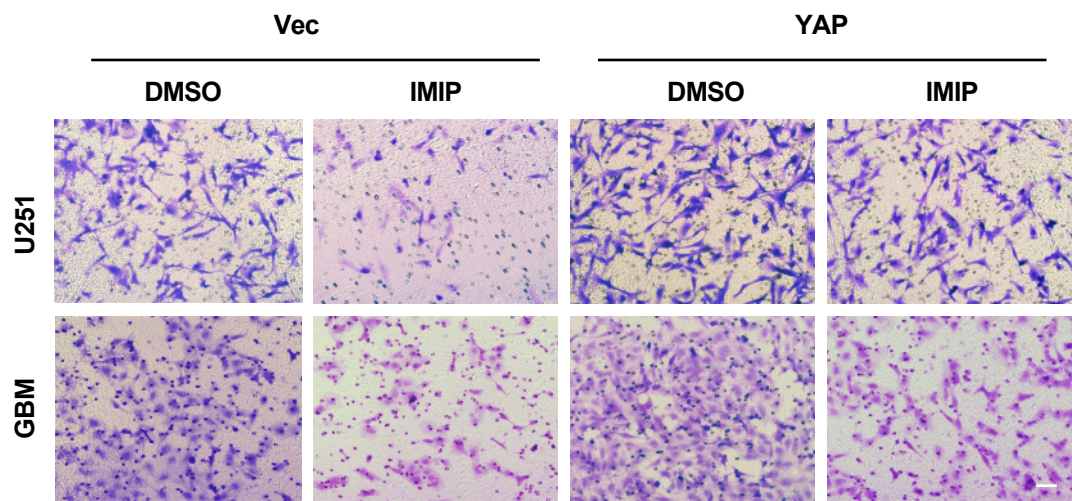

Supplement: Supplementary file 3 — Fig S3 [file JCMM-25-9350-s001.pdf]
