# Supplementary material for: Imipramine impedes glioma progression by inhibiting YAP as a Hippo pathway independent manner and synergizes with temozolomide
Source: J Cell Mol Med. 2021 Sep 1;25(19):9350–63. doi: 10.1111/jcmm.16874 (PMC8500960; doi:10.1111/jcmm.16874)
Supplement: Supplementary file 4 — Supplementary Material [file JCMM-25-9350-s004.docx]

**Supplementary figure legends**

**Figure S1.** **Classification of KEGG annotation of the indicated DEGs by RNA-seq**

192 genes were annotated and enriched in “Environmental Information Processing”, including Signal transduction and Signaling molecules and interaction.

**Figure S2.** **The relative protein levels of endogenous YAP in different cell lines**

Relative YAP protein levels were quantified by YAP/GAPDH ratio in six glioma cells and NHA cell.

**Figure S3.** **YAP partially mediated invasion inhibition of imipramine on glioma cells**

Transwell invasion assay shows that the invasion inhibition effects of imipramine on U251 and GBM cells were restored by YAP-overexpression. Scale bar: 50μm. Data were mean ± SEM for the three replicates. *p < 0.05, **p < 0.01, ***p < 0.001.
